# Proposal of time domain impedance spectroscopy to determine precise dimensionless figure of merit for thermoelectric modules within minutes

**DOI:** 10.1038/s41598-022-15947-4

**Published:** 2022-07-13

**Authors:** Yasuhiro Hasegawa, Mai Takeuchi

**Affiliations:** Graduate School of Science and Engineering, Saitama University, 255, Shimo-okubo, Sakura, Saitama 338-8570 Japan

**Keywords:** Thermoelectrics, Thermoelectric devices and materials, Devices for energy harvesting, Electrical and electronic engineering

## Abstract

Several techniques exist that use a thermoelectric element (TE) or module (TM) to measure precise dimensionless figure of merit (*zT*), both qualitatively and quantitatively. The techniques can be applied using both alternating (AC) and direct current (DC). Herein, the transient Harman (TH) and impedance spectroscopy (IS) methods were investigated as direct *zT* measurement techniques using identical TM, which showed that *zT* at 300 K was 0.767 and 0.811 within several minutes and several hours, respectively. The *zT* values differed despite the use of the same TM, which revealed that measuring ohmic resistance using DC and pulse DC is potentially misleading owing to the influence of Peltier heat on current flow. In this study, time domain impedance spectroscopy (TDIS) was proposed as a new technique to measure *zT* using proper DC and AC. *zT* obtained using TDIS was 0.811 within several minutes using the time and frequency domains, and was perfectly consistent with the result of the IS method. In conclusion, the TDIS is highly appropriate in estimating *zT* directly using only proper electrometric measurements, and without any heat measurements.

## Introduction

Thermoelectric materials and elements (TEs) that can convert a temperature gradient into electricity (Seebeck effect) or electricity into a temperature gradient (Peltier effect) have drawn significant attention as a key technology for renewable energy^1,2^. The performance and energy efficiency of TEs at the temperature *T* have been described as a function of dimensionless figure of merit *zT*, where *z* (= *S*^2^/*ρκ*) is the function of Seebeck coefficient (*S*), resistivity (*ρ*), and thermal conductivity (*κ*)^1–3^. The *zT* values are typically estimated using two different TEs such as a rectangular solid for the measurement of *S* and *ρ*, and a thin disk for the measurement of *κ*^3^; however, the ideal estimation of the *zT* requires the use of the same TE or identical material. A direct *zT* measurement technique using a rectangular solid of the TE was proposed by Harman et al. in 1958. The Harman method^4–6^ uses AC resistance *R*_*AC*_ with an alternating current (AC) and DC resistance *R*_*DC*_ with a direct current (DC), based on which *zT* is expressed as *zT* = *R*_*DC*_/*R*_*AC*_ − 1. However, the applicability of the method is limited owing to lack of information on the frequency of the AC and suitable magnitude of the AC and DC into the TEs.

**Figure 1:**
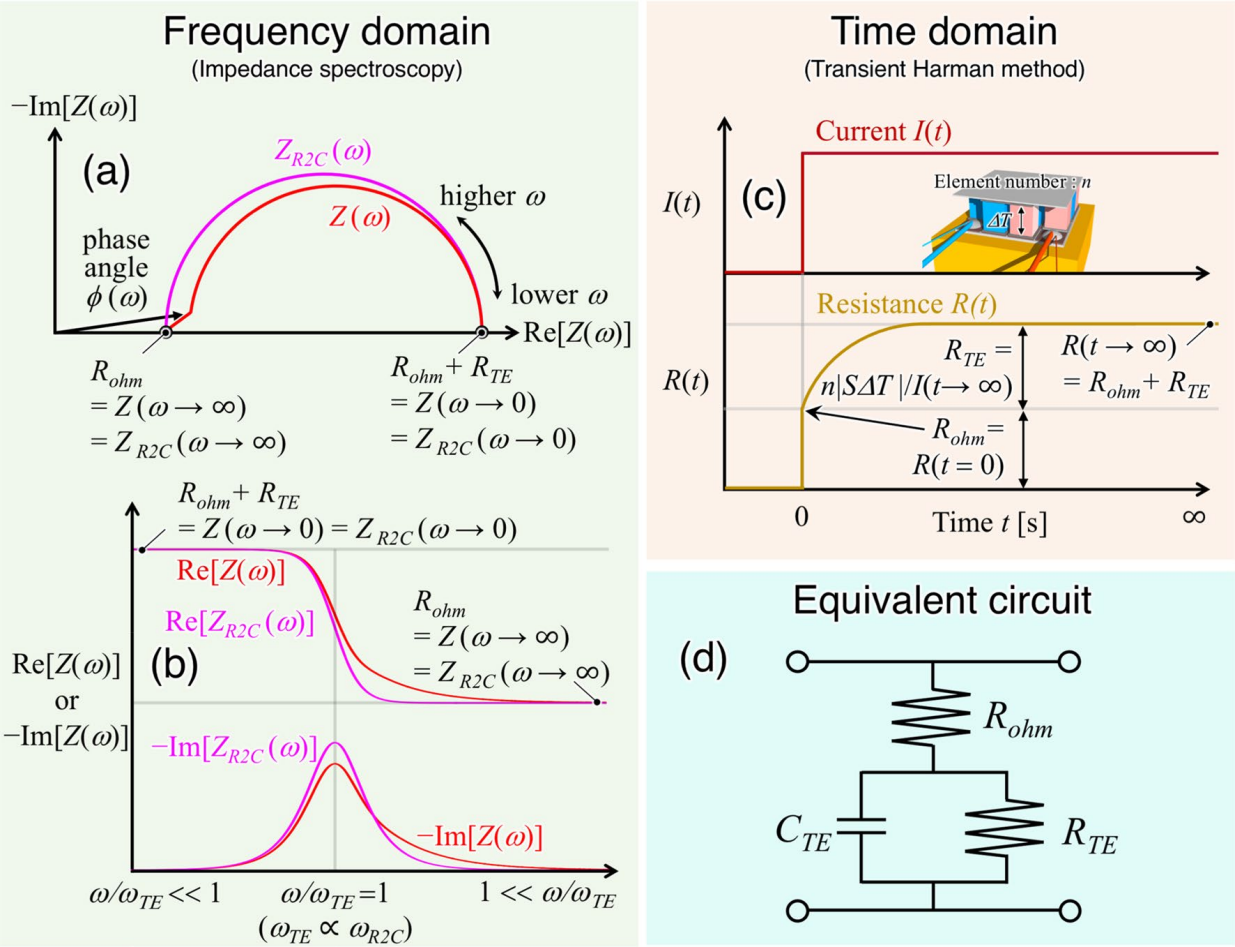
Schematics of (**a**) a Nyquist plot and (**b**) a frequency dependence using the IS method with *Z*(*ω*) and *Z*_*R2C*_(*ω*)^10,12,16^, and (**c**) time dependence of measured resistance *R*(*t*) using the TH method. An inset of (**c**) shows a schematic for the setting view of the TM prepared. (**d**) An equivalent circuit for thermoelectric element and thermoelectric module^16^.

Another approach to directly estimate *zT* using the same TE is a technique called impedance spectroscopy (IS), which uses the frequency domain based on an one-dimensional heat conduction equation^7–16^. Figure 1a,b show the schematic of Nyquist plot and the relation between its angular frequency (*ω*) and impedance *Z*(*ω*), based on which the *zT* is expressed as^10,16^

1 $$zT = \frac{{\left. {Z\left( {\omega \to 0} \right)} \right|_{{Q_{P} > > Q_{J} }} }}{{\left. {Z\left( {\omega \to \infty } \right)} \right|_{{Q_{P} > > Q_{J} }} }} - 1 = \frac{{R_{ohm} + \left. {R_{TE} \left( {1 + \frac{{\eta Q_{J} }}{{Q_{P} }}} \right)} \right|_{{Q_{P} > > Q_{J} }} }}{{R_{ohm} + \left. {\frac{{R_{TE} }}{4}\frac{{Q_{J} }}{{Q_{P} }}} \right|_{{Q_{P} > > Q_{J} }} }} - 1 = \frac{{R_{ohm} + R_{TE} }}{{R_{ohm} }} - 1 = \frac{{R_{TE} }}{{R_{ohm} }} = \frac{{\frac{{S^{2} T}}{\kappa }\frac{L}{A}}}{{\rho \frac{L}{A}}} = \frac{{S^{2} }}{\rho \kappa }T,$$

where *R*_*ohm*_ and *R*_*TE*_ are ohmic and thermoelectric resistance, *Q*_*P*_ ( =|*S|TI*) and *Q*_*J*_ (= *R*_*ohm*_*I*^2^) are Peltier and Joule heat, *A* and *L* are cross- sectional area and length of the TE, respectively, and *η* is a proportional factor associated with the heat flow of *Q*_*J*_ to one side of the TE. In addition, *zT* (= *R*_*TE*_/*R*_*ohm*_) is denoted by the ratio of certain physical parameters such as resistance because *zT* is dimensionless^12,16^. Therefore, the physical meaning of the *zT* implies a ratio of the ohmic resistance (*R*_*ohm*_) to increasing resistance (*R*_*TE*_) generated by the temperature difference (*ΔT*) between the edges of the TE, which, in turn, is caused by the Peltier heat induced by DC. In particular, measuring the resistance, which is a macro physical quantity, is easy using recent electrometric instruments with a combination of a voltmeter for DC measurement and a lock-in amplifier for AC measurement by a precision current source. Equation (1) also shows that the condition *Q*_*P*_ >  > *Q*_*J*_ must be met to obtain the precise value of *zT*. Therefore, the optimum current *I*_*opt*_ should be *I*_*opt*_ < <|*S*|*T*/*R*_*ohm*_. Moreover, the IS method is a suitable technique to determine the *zT* for both TEs and thermoelectric modules (TMs), which are an assembly of thermoelectric elements^10,11,16^. However, measuring the *Z*(*ω* → 0) takes several hours because a suitable characteristic frequency (*ω*_*TE*_) is required at *ω* → 0 (or *ω* <  < *ω*_*TE*_). *ω*_*TE*_ is denoted as a function of thermal diffusivity (*α*) and *L* of the TE, *ω*_*TE*_ ∝ *α*/*L*^2^, and typically approaches 1 rad/s owing to the small value of *κ* (∝ *α*) of the TEs. Therefore, the angular frequency satisfying *Z*(*ω* → 0) would approximately be of the order of 10^–2^–10^–4^ rad/s, depending on *L*^12,15^. The theory and model of the IS method clearly demonstrates that the Harman method is one of the results obtained using *Z*(*ω* → ∞) → *R*_*AC*_ and *Z*(*ω* → 0) → *R*_*DC*_ from Eq. (1) at *Q*_*P*_ >  > *Q*_*J*_^16^. Furthermore, the R2C approximation was applied to roughly explain the angular frequency dependence *Z*_*R2C*_(*ω*), as shown in Fig. 1a,b, using optimum current *I*_*opt*_, which is expressed as^12,16^

2 $$Z_{R2C} \left( \omega \right) = R_{ohm} + \left. {R_{TE} \left( {1 + \frac{{\eta Q_{J} }}{{Q_{P} }}} \right)} \right|_{{Q_{P} > > Q_{J} }} \left( {\frac{{1 - \left( {{{j\omega } \mathord{\left/ {\vphantom {{j\omega } {\omega_{R2C} }}} \right. \kern-\nulldelimiterspace} {\omega_{R2C} }}} \right)}}{{1 + \left( {{\omega \mathord{\left/ {\vphantom {\omega {\omega_{R2C} }}} \right. \kern-\nulldelimiterspace} {\omega_{R2C} }}} \right)^{2} }}} \right)= R_{ohm} + R_{TE} \left( {\frac{{1 - \left( {{{j\omega } \mathord{\left/ {\vphantom {{j\omega } {\omega_{R2C} }}} \right. \kern-\nulldelimiterspace} {\omega_{R2C} }}} \right)}}{{1 + \left( {{\omega \mathord{\left/ {\vphantom {\omega {\omega_{R2C} }}} \right. \kern-\nulldelimiterspace} {\omega_{R2C} }}} \right)^{2} }}} \right)$$

The transient Harman (TH) method, which is an alternative technique derived from the Harman method, is based on the transient response of resistance *R*(*t*) of the TEs and TMs using time domain, as shown in Fig. 1c. The TH method is relatively simpler to apply in the determination of *zT* using results of the *R*(*t*); several researchers have also reported its applicability^17–22^. In this method, *R*_*ohm*_ and *R*_*ohm*_ + *R*_*TE*_ correspond to *R*(*t* = 0) and *R*(*t* → ∞), respectively, and *zT* is expressed as *zT* = *R*(*t* → ∞)/*R*(*t* = 0) − 1 using Eq. (1). The IS and TH methods (or R2C approximation) show that an equivalent circuit of the TE and TM can be expressed using three components, namely, *R*_*ohm*_, *R*_*TE*_, and *C*_*TE*_ (called thermoelectric capacity), as shown in Fig. 1d. *C*_*TE*_ is related to heat capacity of not only the TE(s) but also other components constituting the TE(s) like electrodes, especially for the TM^10,12,16^.

In this study, we comprehensively investigate several techniques of direct *zT* estimation based on the IS and TH methods. We employ a commercial base Π-shaped TM composed of bismuth-telluride (BiTe) (inset in Fig. 1c) to avoid influences of heat leakages through lead-wires attached for measurement and their contact resistance^23^. Furthermore, we highlight the disadvantages of both methods and suggest new techniques to overcome the drawbacks of the conventional methods. Finally, we propose a suitable technique to determine the value of *zT* precisely and directly within several minutes using a combination of AC and DC electrometric instruments, called time domain impedance spectroscopy (TDIS), and discuss the important factors required to obtain the value of *zT* precisely through measurements.

## Results

**Figure 2:**
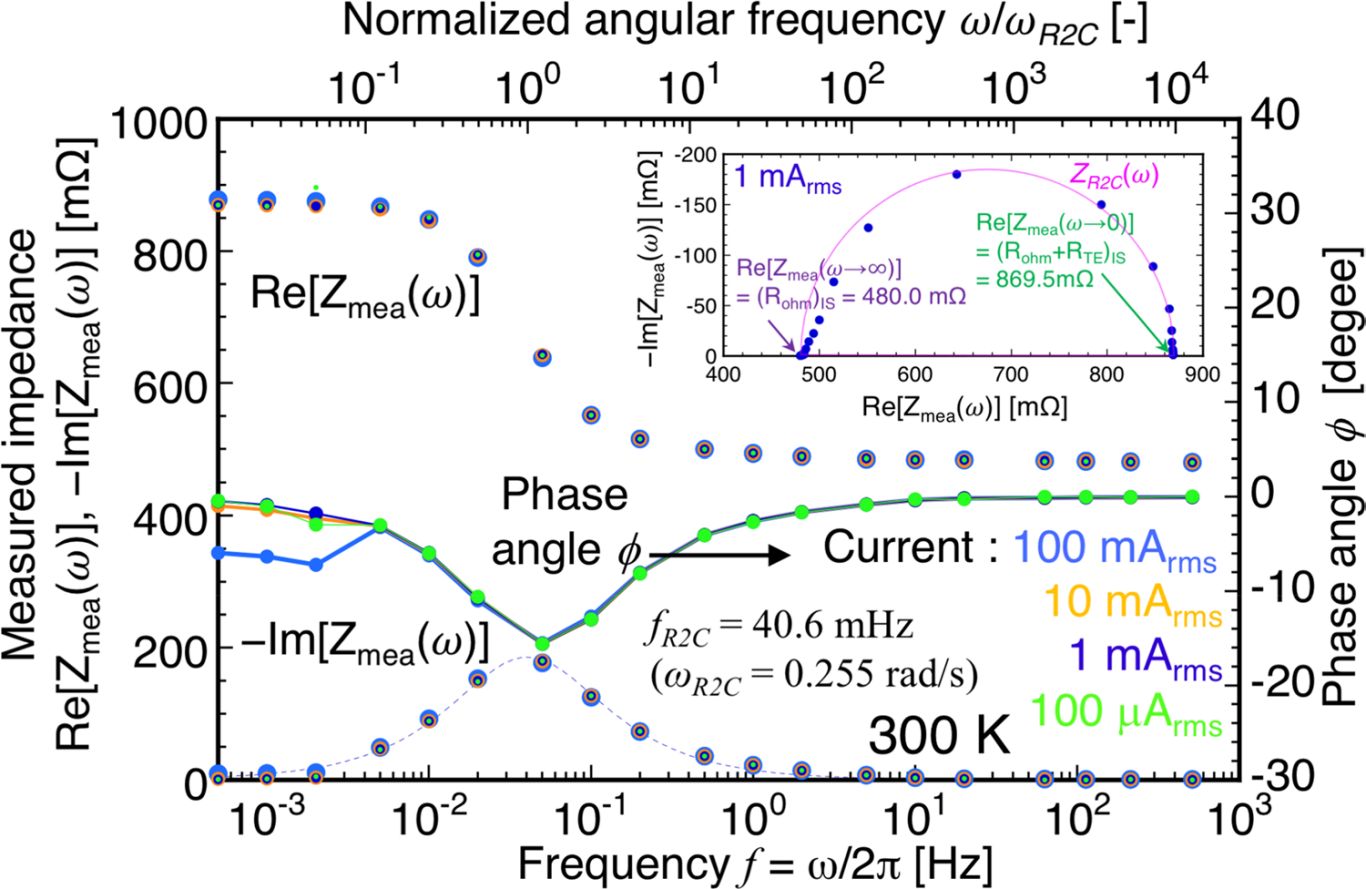
Frequency dependence of impedance of real Re[*Z*_*mea*_(*ω*)] and imaginary part − Im[*Z*_*mea*_(*ω*)], respectively and phase angle *ϕ* at each AC (100 μA_rms_ to 100 mA_rms_) for the TM prepared. The upper axis shows normalized angular frequency *ω*/*ω*_*R2C*_. A lock-in amplifier and Quasi-AC method (implemented using a high-precision AC source and digital multimeter using real-time data acquisition for the low-frequency region)^12,28^ were applied to measure the impedances at frequencies more than and less than 10 mHz, respectively. An inset shows its Nyquist plot of *Z*_*R2C*_(*ω*) and fitting plot by R2C approximation given in Eq. (2).

Figure 2 shows the frequency dependence of the measured impedance *Z*_*mea*_(*ω*) in the case of the IS method for a TM prepared at 300 K with various AC from *I* = 100 μA_rms_ to 100 mA_rms_^16^, and the inset shows its Nyquist plot using 1 mA_rms_. The characteristic angular frequency (*ω*_*R2C*_) using the R2C approximation given by Eq. (2) was 0.255 rad/s (= 40.6 mHz) owing to *L* = 1.4 mm (*ω*_*R2C*_ ∝ 1/*L*^2^). (*R*_*ohm*_)_IS_ = *Z*_*mea*_(*ω* → ∞) = Re[*Z*_*mea*_(*f* = 513 Hz)] at *ω*/*ω*_*R2C*_ ~ 10^4^ (or phase angle |*ϕ*|< 0.1°) was 480.0 mΩ, and (*R*_*ohm*_ + *R*_*TE*_)_IS_ = *Z*_*mea*_(*ω* → 0) = Re[*Z*_*mea*_(*f* = 0.5 mHz)] at *ω*/*ω*_*R2C*_ ~ 10^–2^ (or |*ϕ*|< 0.1°) was 869.5 mΩ at a current less than 10 mA_rms_, satisfying *Q*_*P*_ >  > *Q*_*J*_. At 100 mA_rms_, the contribution of *Q*_*J*_ affected Re[*Z*_*mea*_(*ω*)] and − Im[*Z*_*mea*_(*ω*)] in the lower frequency region. Therefore, Re[*Z*_*mea*_(*ω*)] (∝ *I*) increased marginally at 10^–3^ Hz owing to *Q*_*P*_ (= 6.9 mW) ~ *Q*_*J*_ (= 4.8 mW) using the representative magnitude of *S* (= − 231 μV/K at 300 K) corresponding to BiTe standard material^24^. In addition, *ϕ* = tan^−1^(Im[*Z*_*mea*_(*ω*)]/Re[*Z*_*mea*_(*ω*)]) helps identify the suitable frequency for determining *Z*_*mea*_(*ω* → ∞) and *Z*_*mea*_(*ω* → 0) because *ω*_*R2C*_ is unknown^15^. Finally, using the IS method, the value of *zT* was clearly estimated as (*zT*)_IS_ = 0.811 (= (*R*_*ohm*_ + *R*_*TE*_)_IS_/(*R*_*ohm*_)_IS_ − 1 = 869.5 / 480.0 − 1).

**Figure 3:**
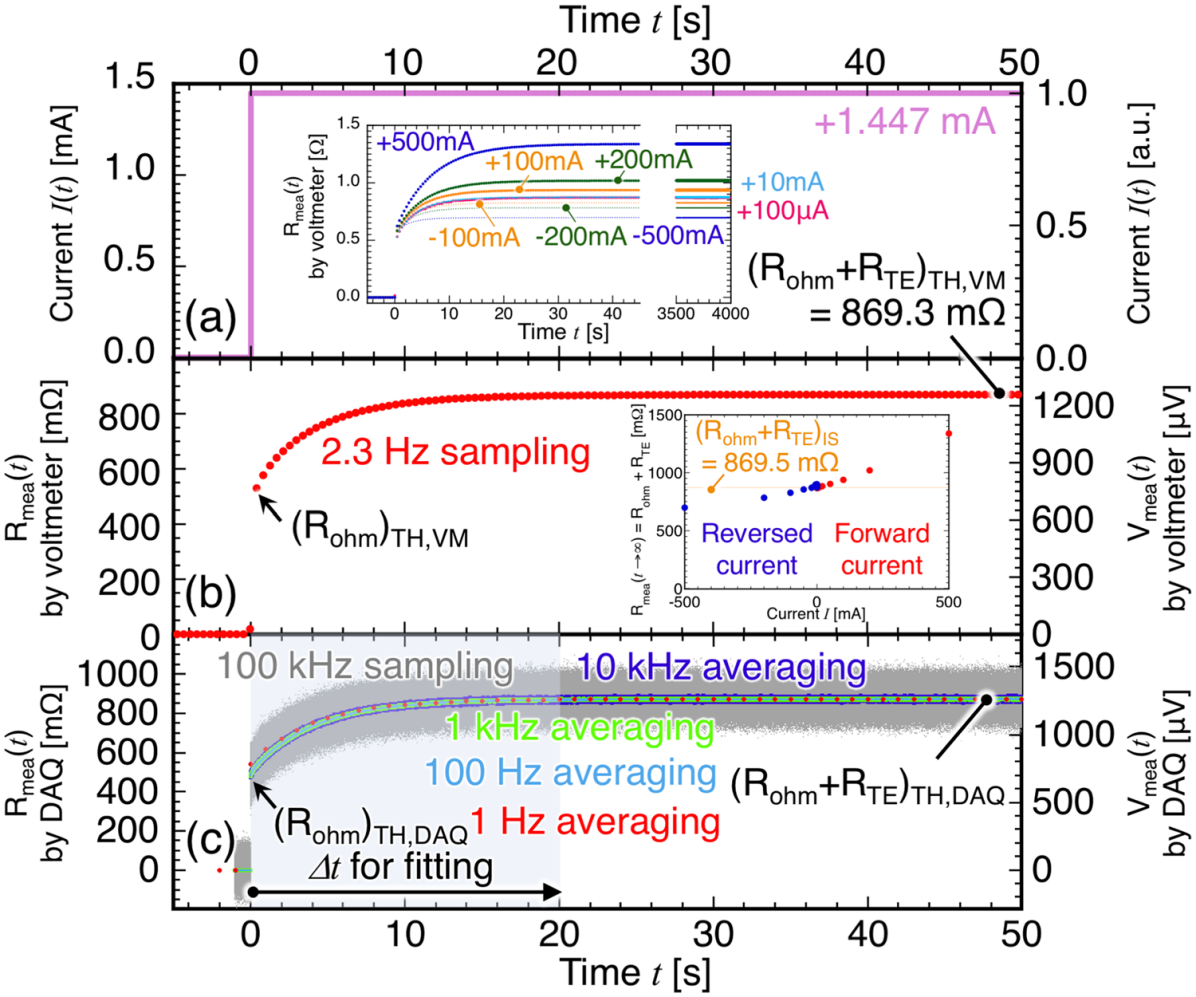
Time dependence of transient response for (**a**) direct current *I*(*t*), measured resistance *R*_*mea*_(*t*) and voltage *V*_*mea*_(*t*) by (**b**) a voltmeter, and (**c**) a DAQ system at a sampling rate of using 100 kHz and its averaging results without error bar for each averaging period, respectively. The insets of (**a**) and (**b**) show the time dependence of *R*_*mea*_(*t*) at each current and current dependence of *R*_*mea*_(*t* → ∞), respectively.

Figure 3 shows the results of the TH method for the same TM used in the IS method at 300 K for transient current *I*(*t*). *R*_*mea*_(*t*) was measured using a voltmeter (VM) and the remaining measurements were made using a data acquisition (DAQ) system. The inset in Fig. 3a shows the time dependence of *R*_*mea*_(*t*) for different values of current ranging from − 500 mA to + 500 mA. In addition, the current dependencies of *R*_*mea*_(*t* → ∞) at higher magnitudes of current due to the influence of *Q*_*J*_ are clearly observable. *I*(*t*) using 1.447 mA at *t* ≥ 0 that is shown in Fig. 2a was selected from the essential condition of *Q*_*P*_ (= 100 μW) >  > *Q*_*J*_ (= 1 μW) using (*R*_*ohm*_)_IS_. Figure 2b shows that the value of *R*_*mea*_(*t* → ∞) = (*R*_*ohm*_ + *R*_*TE*_)_TH,VM_ = 869.3 mΩ measured by the voltmeter was specifically determined owing to the large signal-to-noise ratio (SNR). *R*_*mea*_(*t* → ∞) was also measured at a different range of current, as shown in an inset in Fig. 3b, because *R*_*TE*_ was replaced with *R*_*TE*_(1 + *ηQ*_*J*_/*Q*_*P*_) in the higher current region in Eq. (1). It shows that *R*_*mea*_(*t* → ∞) is proportional to *I* (∝ *Q*_*J*_/*Q*_*P*_) in the higher current region, as expected^16^. To avoid aliasing of the signals measured by the voltmeter, the sampling rate was set as 2.3 Hz; consequently, *R*_*mea*_(*t* → 0) = (*R*_*ohm*_)_TH,VM_ was ambiguous. Therefore, data acquisition had to be performed at a higher sampling rate to detect the variation of *R*_*mea*_(*t* → 0). The characteristic frequency *ω*_*R2C*_ = 0.255 rad/s can be used as an approximate standard to derive the heat time constant *τ*_*exp*_ of the system (~ 4 s = 1/*ω*_*R2C*_). Figure 3c also shows *R*_*mea*_(*t*) obtained using a DAQ system at a sampling rate of 100 kHz, which would be sufficient to detect the transient response of *R*_*mea*_(*t*). Owing to the small SNR in the surroundings (or higher sampling rate), the data was averaged for a period ranging from 10 kHz to 1 Hz. However, detecting *R*_*mea*_(*t* = 0) from the results of the TH method is difficult despite the use of the DAQ. As the expected value of *R*_*mea*_(*t* = 0) was 480.0 mΩ from (*R*_*ohm*_)_IS_, the first data for *R*_*mea*_(*t* = 0) was 471.27, 477.57, and 483.32 mΩ at 10 kHz, 1 kHz, and 100 Hz averaging, respectively. At a sampling rate of 100 kHz, the raw data of *R*_*mea*_(*t* → 0) was distributed from 23.94 to 554.62 mΩ during 10 μs. This result shows that detecting *R*_*ohm*_ using the raw data of the TH method is difficult despite using the higher DAQ system.

**Figure 4:**
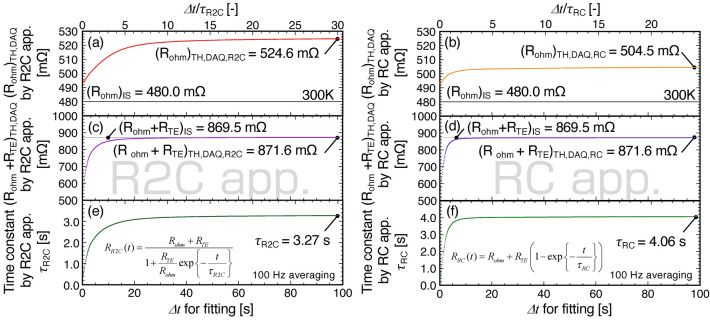
Estimated (**a**,**b**) (*R*_*ohm*_)_TH,DAQ_ (= *R*_*mea*_(*t* = 0)), (**c**,**d**) (*R*_*ohm*_ + *R*_*TE*_)_TH,DAQ_ (= *R*_*mea*_(*t* → ∞)), and (**e**,**f**) time constant (*τ*_*R2C*_ and *τ*_*RC*_) by R2C and RC approximation using the Eqs. (3) and (4) for the period *Δt* in Fig. 3c using the DAQ system (100 Hz averaging), respectively. The upper axes show the normalized time *Δt*/*τ*_*R2C*_ or *Δt*/*τ*_*RC*_, respectively.

Another method was developed to determine *R*_*ohm*_ and *R*_*ohm*_ + *R*_*TE*_ by fitting the formula into the data obtained by the DAQ system. This proposal was based on the fact that *R*_*mea*_(*t*) is almost stabilized at 100 Hz averaging. Therefore, we used the value of *R*_*mea*_(*t*) measured by the DAQ using 100 Hz averaging in the TH method. This approach was adopted based on our hypothesis that the average frequency enables us to represent the transient response with *τ*_*exp*_. To estimate *R*_*mea*_(*t* → 0) = (*R*_*ohm*_)_TH,DAQ_ and *R*_*mea*_(*t* → ∞) = (*R*_*ohm*_ + *R*_*TE*_)_TH,DAQ_ from Fig. 3c, two fitting equations for period *Δt* were applied from the equivalent circuit in Fig. 1d ^16^.

3 $$R_{R2C} (t) = \frac{{R_{ohm} + \left. {R_{TE} \left( {1 + \frac{{\eta Q_{J} }}{{Q_{P} }}} \right)} \right|_{{Q_{p} > > Q_{J} }} }}{{1 + \left. {\frac{{R_{TE} }}{{R_{ohm} }}\left( {1 + \frac{{\eta Q_{J} }}{{Q_{P} }}} \right)} \right|_{{Q_{p} > > Q_{J} }} \exp \left\{ { - \frac{t}{{\tau_{R2C} }}} \right\}}} = \frac{{R_{ohm} + R_{TE} }}{{1 + \frac{{R_{TE} }}{{R_{ohm} }}\exp \left\{ { - \frac{t}{{\tau_{R2C} }}} \right\}}},$$

4 $$R_{RC} (t) = R_{ohm} + \left. {R_{TE} \left( {1 + \frac{{\eta Q_{J} }}{{Q_{P} }}} \right)} \right|_{{Q_{p} > > Q_{J} }} \left( {1 - \exp \left\{ { - \frac{t}{{\tau_{RC} }}} \right\}} \right) = R_{ohm} + R_{TE} \left( {1 - \exp \left\{ { - \frac{t}{{\tau_{RC} }}} \right\}} \right),$$

where *τ*_*R2C*_ and *τ*_*RC*_ are the estimated time constants using each equation, respectively. Figure 4 shows the calculation results from the period *Δt*. Figure 4a,b show *R*_*mea*_(*t* → 0) using the R2C (= (*R*_*ohm*_)_TH,DAQ,R2C_) and RC (= (*R*_*ohm*_)_TH,DAQ,RC_) approximations expressed in Eqs. (3) and (4), respectively. Near *Δt* ~ 0, both (*R*_*ohm*_)_TH,DAQ,R2C_ in Fig. 4a and (*R*_*ohm*_)_TH,DAQ,R2C_ in Fig. 4b were approximately 494 mΩ, and increased with increasing *Δt* owing to the excessive data available for *R*_*mea*_(*t*). Finally, (*R*_*ohm*_)_TH,DAQ,R2C_ and (*R*_*ohm*_)_TH,DAQ,RC_ were asymptotically close to 524.6 mΩ in Fig. 4a and 504.5 mΩ in Fig. 4b, respectively, which are approximately 1.09 and 1.05 times higher compared with (*R*_*ohm*_)_IS_ = 480.0 mΩ. The difference between (*R*_*ohm*_)_TH,DAQ_ and (*R*_*ohm*_)_IS_ obstructs the determination of (*R*_*ohm*_)_TH,DAQ_ when estimating *zT*. In other words, the value of *zT* is underestimated because (*R*_*ohm*_)_TH,DAQ_ > (*R*_*ohm*_)_IS_. Therefore, estimating (*R*_*ohm*_)_TH,DAQ_ from *R*_*mea*_(*t* → 0) obtained using the TH method is unsuitable even if the DAQ was used for measurement. Furthermore, Fig. 4c,d show that (*R*_*ohm*_ + *R*_*TE*_)_TH,DAQ_ converged at certain values, i.e., (*R*_*ohm*_ + *R*_*TE*_)_TH,DAQ,R2C_ = (*R*_*ohm*_ + *R*_*TE*_)_TH,DAQ,RC_ = 871.6 mΩ in Fig. 4c,d, which are consistent with (*R*_*ohm*_ + *R*_*TE*_)_IS_ = 869.5 mΩ. The difference is acceptable because the difference is approximately 2.1 mΩ, which corresponds to the approximate 3 μV difference during the DC measurement. Moreover, the estimated time constants *τ*_*R2C*_ and *τ*_*RC*_ were 3.27 and 4.06 s, respectively, neither of which matched the estimated value. However, in the RC approximation using Eq. (4), *τ*_*RC*_ = 4.06 s is not only close to *τ*_*exp*_ = 1/*ω*_*R2C*_ = 4 s as a representative heat transport time in this system, but also simpler to apply. Figure 4f quantitatively shows that the required period using the normalized time *Δt*/*τ*_*RC*_ is more than 10 for each parameter. Furthermore, (*zT*)_TH,DAQ_ was estimated to be 0.767 ± 0.014 (= (*R*_*ohm*_ + *R*_*TE*_)_TH,DAQ,RC_/(*R*_*ohm*_)_TH,DAQ,RC_ − 1 = 871.6/504.5 − 1), which is marginally smaller than that of the IS method because it is likely to overestimate (*R*_*ohm*_)_TH,DAQ_ compared with (*R*_*ohm*_)_IS_.

**Figure 5:**
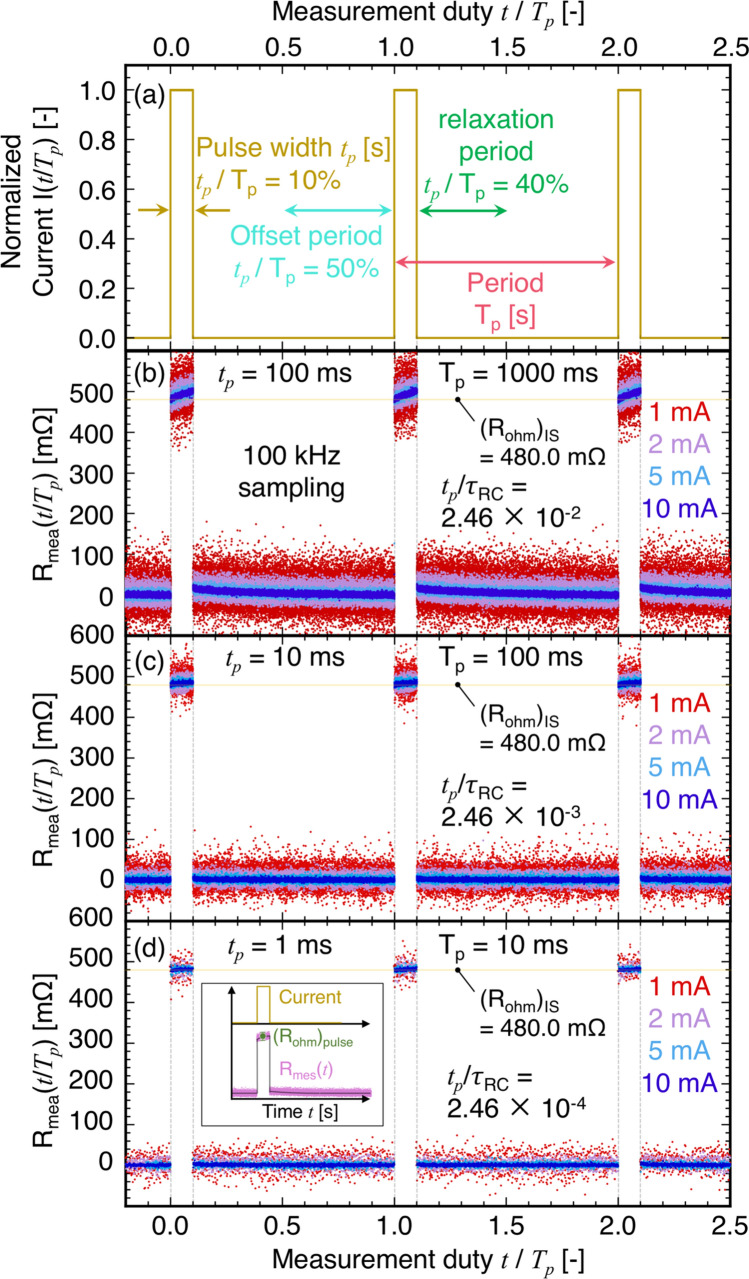
Normalized time dependence of (**a**) pulse current *I*(*t*/*T*_*p*_), measured resistance *R*_*mea*_(*t*/*T*_*p*_) at (**b**) *t*_*p*_ = 100 ms and *T*_*p*_ = 1000 ms, (**c**) *t*_*p*_ = 10 ms and *T*_*p*_ = 100 ms, (**d**) *t*_*p*_ = 1 ms and *T*_*p*_ = 10 ms for each pulse DC (1–10 mA ) by a DAQ system (100 kHz sampling rate), respectively. 10%, 40%, and 50% of the entire pulse period *T*_*p*_ in (**a**) correspond to the pulse DC width *t*_*p*_, relaxation period to remove the temperature gradient (or difference) on the TEs of the TM, and offset period to determine the zero voltage for the next measurement, respectively. The inset in (**d**) shows how (*R*_*ohm*_)_pulse_ can be estimated from obtained data.

We also attempted to precisely measure *R*_*ohm*_ using the pulse DC with an identical DAQ system because the *R*_*ohm*_ term adds the contribution of the temperature difference generated for the DC. Figure 5 shows the results of measuring *R*_*mea*_(*t* → 0) as a function of *t*/*T*_*p*_, where *T*_*p*_ is the pulse period. From Eqs. (1) and (4), the expression for *R*_*mea*_(*t* → 0) at *Q*_*P*_ >  > *Q*_*J*_ can be derived as

5 $$R_{mea} (t \to 0) \simeq R_{RC} (t \to 0) = R_{ohm} + R_{TE} \left( {1 - \exp \left\{ { - \frac{t}{{\tau_{RC} }}} \right\}} \right) \simeq R_{ohm} + R_{TE} \frac{t}{{\tau_{RC} }} = R_{ohm} \left( {1 + zT\frac{t}{{\tau_{RC} }}} \right).$$

Although the values of *R*_*mea*_(*t*/*T*_*p*_ = 0) were approximately 480.0 mΩ (= (*R*_*ohm*_)_IS_) within the scattered data, it increased linearly at *t*_*p*_ = 100 ms due to *t*_*p*_/*τ*_*RC*_ = 2.46 × 10^–2^, as expected from Eq. (5). Moreover, if the value of *zT* is large, typically 1, the term *zT* × *t*_*p*_/*τ*_*RC*_ in Eq. (5) should be less than 10^–3^ to ensure precise *R*_*ohm*_ measurement. The increase in *R*_*mea*_(*t*/*T*_*p*_ ~ 0) was suppressed at shorter *t*_*p*_, and the variation of *R*_*mea*_(*t*) during *t*_*p*_ was considered negligible owing to the considerably lower *t*_*p*_/*τ*_*RC*_, as shown in Fig. 5d for *t*_*p*_/*τ*_*RC*_ = 2.46 × 10^–4^. Furthermore, current dependence was absent owing to a shorter *t*_*p*_, which possibly satisfies *Q*_*P*_ >  > *Q*_*J*_, during the *t*_*p*_/*T*_*p*_ cycle. Finally, although the use of pulse DC was expected to be suitable for measuring (*R*_*ohm*_)_pulse_, the measurement error within the scattered data near *t*/*T*_*p*_ ~ 0 was also considered.

**Figure 6:**
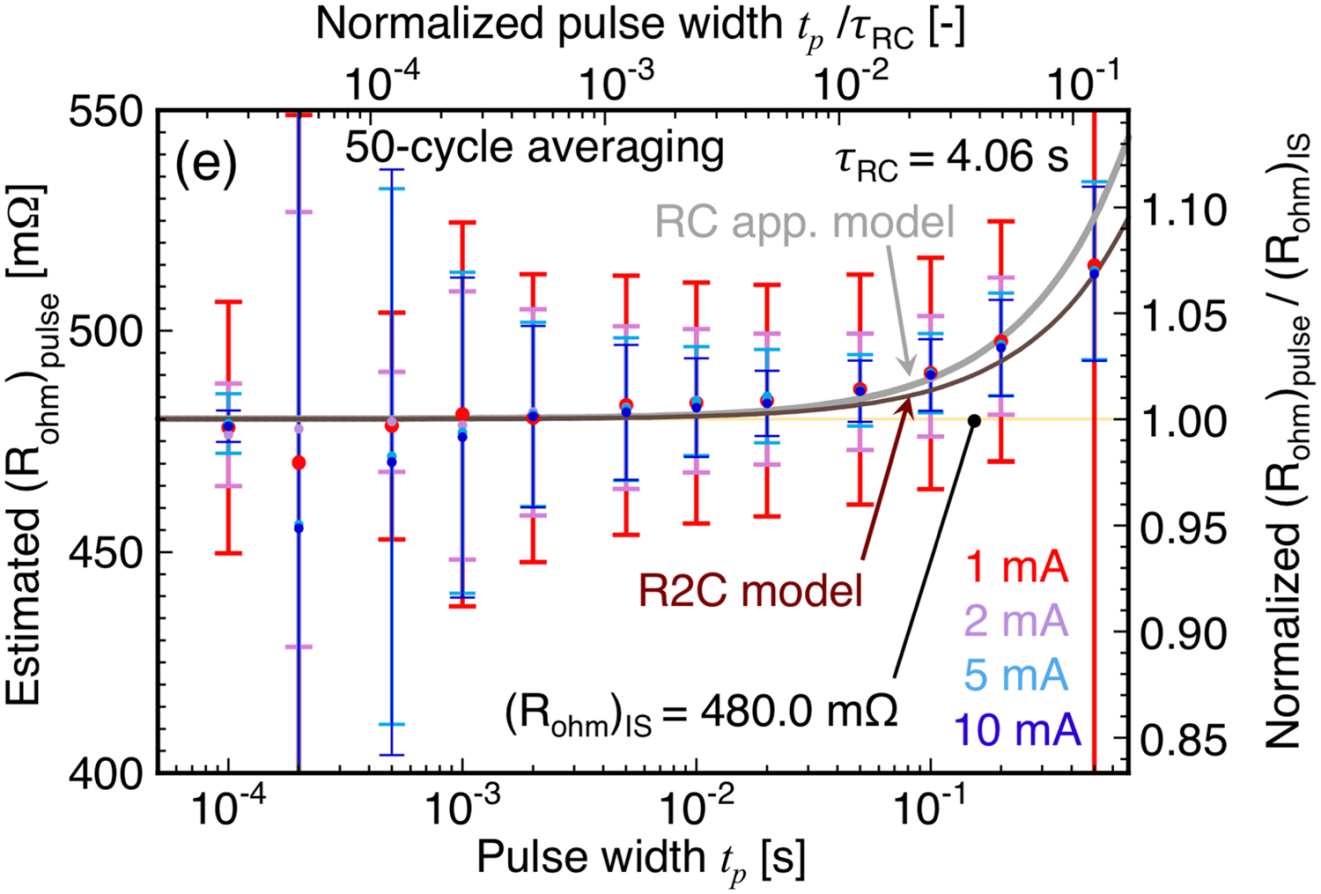
Pulse width (*t*_*p*_) dependence of estimated ohmic resistance (*R*_*ohm*_)_pulse_ using 50-cycle averaging at each current. The upper axis shows normalized pulse width *t*_*p*_/*τ*_*RC*_. The results using the R2C and RC approximation models with (*R*_*ohm*_)_IS_ are also shown.

Figure 6 shows the *t*_*p*_ dependence of (*R*_*ohm*_)_pulse_, and fell within the accepted error margin. It quantitatively shows that (*R*_*ohm*_)_pulse_ approached (*R*_*ohm*_)_IS_ = 480.0 mΩ at *t*_*p*_/*τ*_*RC*_ < 10^–3^, as expected from Eq. (5). However, the measurement error of (*R*_*ohm*_)_pulse_ was prominent at all *t*_*p*_ owing to the low SNR. Moreover, (*R*_*ohm*_)_pulse_ measured at a larger current, i.e., 10 mA, resulted in a smaller error compared with that measured at a smaller current; however, the error in the measurement of (*R*_*ohm*_)_pulse_ was still large. Based on this evaluation, pulse and transient Harman (PTH) method was proposed for determining (*zT*)_PTH_ using (*R*_*ohm*_)_pulse_ and (*R*_*ohm*_ + *R*_*TE*_)_TH,DAQ_. (*zT*)_PTH_ was estimated to be 0.804 ± 0.043 (= (*R*_*ohm*_ + *R*_*TE*_)_TH,DAQ_/(*R*_*ohm*_)_pulse_ − 1 = 871.6/483.2 − 1) using (*R*_*ohm*_)_pulse_ = 483.2 mΩ at *I* = 1 mA and *t*_*p*_ = 5 ms (*t*_*p*_ /*τ*_*RC*_ ~ 10^–3^), which is close to that of the IS method. Additionally, we attempted to measure (*R*_*ohm*_)_delta_ using the delta method, in which a current source is synchronized with an oscillating square wave at a frequency (a kind of pulse current) using a voltmeter^25^. Subsequently, the resistance can be measured after eliminating offset voltage and removing the influences of the thermoelectric-motive force in the circuit. The details are summarized in Supplementary Information. Furthermore, an important conclusion was drawn from the measurements that were used to determine (*R*_*ohm*_)_pulse_ using pulse DC in Figs. 5 and 6, (*R*_*ohm*_)_delta_ in Fig. S1, and *R*_*ohm*_ + *R*_*TE*_ using DC in Fig. 3c by the PTH method. The precise measurement of *R*_*ohm*_ from *R*_*mea*_(*t* → 0) using only continuous DC is unsuitable for the TMs and TEs possessing large *zT* values despite the use of the DAQ system. This unsuitability can be attributed to the fact that large *zT* values affect the measurement noise (depending on the surroundings in the measurement system) even if the normalized pulse width *t*_*p*_/*τ*_*RC*_ is less than 10^–3^. Although the transported Peltier heat *Q*_*P*_ at *I* = 1 mA, shown Figs. 5 and 6 appeared small near *Q*_*P*_ = 69.3 μW ( =|*S*|*TI* = 231 × 10^–6^ × 300 × 1 × 10^–3^) at 300 K^24^, the Peltier heat flux *q*_*P*_ across the TE was not negligible, i.e., *q*_*P*_ = 164 W/m^2^ (= *Q*_*P*_/*A* = 69.3 × 10^–6^ × (0.65 × 10^–3^)^−2^) for *A* = 0.65 × 0.65 mm^2^ owing to the generation of *ΔT* (~ *q*_*p*_*L*/*κ*).

## Discussion

Based on the above-mentioned results and considerations, we proposed a combined measurement technique, namely, time domain impedance spectroscopy (TDIS), which attempts to measure *R*_*ohm*_ = *Z*_*mea*_(*ω* → ∞) using a lock-in amplifier and AC at *ω*/*ω*_*R2C*_ > 10^4^ (or |*ϕ*|< 0.1°) and *R*_*ohm*_ + *R*_*TE*_ = *R*_*mea*_(*t* → ∞) with a voltmeter. Alternatively, it employs a DAQ system using DC at *t*/*τ*_*RC*_ > 10 based on the TH method and partially derives the optimum current from the knowledge of the IS theory and model^10,11,16^. Consequently, (*zT*)_TDIS_ was expressed as (*zT*)_TDIS,VM_ = 0.811 ± 2.4 × 10^–4^ (= (*R*_*ohm*_ + *R*_*TE*_)_TH,VM_/(*R*_*ohm*_)_IS_ − 1 = 869.3/480 − 1) using the voltmeter or (*zT*)_TDIS,DAQ_ = 0.816 ± 3.6 × 10^–4^ (= (*R*_*ohm*_ + *R*_*TE*_)_TH,DAQ,RC_/(*R*_*ohm*_)_IS_ − 1 = 871.6/480 − 1) using the DAQ (data of 100 Hz averaging). A marginal difference between (*zT*)_TDIS,VM_ and (*zT*)_TDIS,DAQ_ was derived from the measurement error of (*R*_*ohm*_ + *R*_*TE*_)_TH,VM_ and (*R*_*ohm*_ + *R*_*TE*_)_TH,DAQ,RC_ depending on SNR. The magnitude of the current *I*_*max*_(*t* > 0) required to suitably measure *Z*_*mea*_(*ω* > 10^4^ *ω*_*R2C*_) and *R*_*mea*_(*t* > 10*τ*_*RC*_) should fulfil the condition *I*_*max*_ <  < *R*_*ohm*_/(*n*|*S*|*T*) to satisfy *Q*_*P*_ >  > *Q*_*J*_, where *n* is number of TEs in the TM (*n* = 1 is for the TE). In short, although its concept is relatively similar to that of the original Harman method^4^, the TDIS method accidentally revealed the method to measure *Z*_*mea*_(*ω* → ∞) and *R*_*mea*_(*t* → ∞) both quantitatively and qualitatively. Additionally, the temperature fluctuation *ΔT*_*f*_ of the sample stage anchoring the TM during the measurements is an important factor. This *ΔT*_*f*_ influences the measurement of the additional *R*_*TE*_ (= *n|SΔT|*/*I*) from *R*_*ohm*_ because the TDIS method is based on detecting *ΔT* derived from the Peltier effect (*ΔT* = *R*_*TE*_*I*/*n*|*S*|), given that *ΔT* >  > *ΔT*_*f*_. Furthermore, all the experiments in this study were performed at a standard deviation of *ΔT*_*f*_ ~ 0.3 mK in high vacuum (~ 10^–4^ Pa) to maintain an adiabatic condition^26,27^. We hypothesize that *ΔT*_*f*_/*ΔT* (~ 0.3 mK/174 mK) is a key factor that determines how accurately the value of *zT* can be detected by the TDIS^24^.

**Figure 7:**
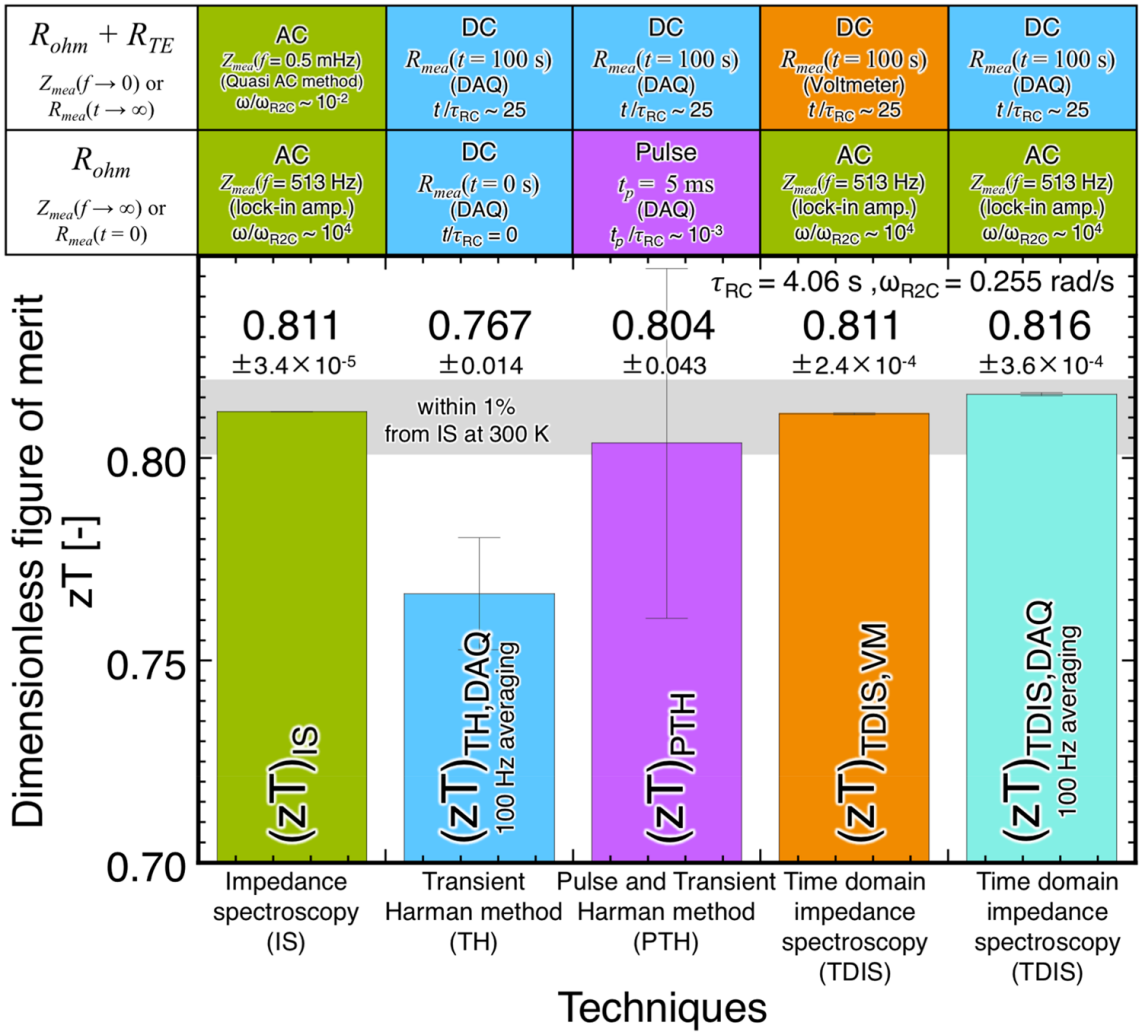
A summary of *zT* of the prepared TM estimated by various techniques, devices used, and conditions for *R*_*ohm*_ and *R*_*ohm*_ + *R*_*TE*_ at 300.000 K.

Figure 7 and Table 1 show a summary of *zT* estimation using each technique. The (*zT*)_IS_ estimated using the IS method, as shown in Fig. 2, was 0.811 ± 3.4 × 10^–5^. The result is reliable and (*R*_*ohm*_)_IS_ (= Re[*Z*_*mea*_(*f* = 513 Hz)]) was obtained by the lock-in amplifier within several seconds; however, the measurement of (*R*_*ohm*_ + *R*_*TE*_)_IS_ (= Re[*Z*_*mea*_(*f* = 0.5 mHz)]) required several hours when employing the quasi-AC method in the lower frequency region (< 10 mHz) using the DC source and the voltmeter^12,28^. (*zT*)_TH,DAQ_ estimated using the TH method, as shown in Figs. 3 and 4, was 0.767 ± 0.014. The estimated (*zT*)_TH,DAQ_ differed from (*zT*)_IS_ because determining (*R*_*ohm*_)_TH,DAQ_ (= *R*_*mea*_(*t* → 0)) was difficult despite applying the DAQ system. Finally, (*zT*)_PTH_ estimated using the PTH method with pulse DC, as shown in Figs. 4, 5, and 6, was 0.804 ± 0.043, which was in moderately good agreement with (*zT*)_IS_. However, the measurement error to determine (*R*_*ohm*_)_pulse_ was prominent. In the TDIS method, to quickly determine *R*_*ohm*_ and *R*_*ohm*_ + *R*_*TE*_ without error, (*R*_*ohm*_)_IS_ and (*R*_*ohm*_ + *R*_*TE*_)_TH,VM_ or (*R*_*ohm*_ + *R*_*TE*_)_TH,DAQ_ obtained from the lock-in amplifier and the voltmeter or the DAQ using the TH method with suitable AC and DC, respectively, were used. The *zT* values estimated by the voltmeter and the DAQ were (*zT*)_TDIS,VM_ = 0.811 ± 2.4 × 10^–4^ and (*zT*)_TDIS,DAQ_ = 0.816 ± 3.6 × 10^–4^, respectively. In addition, the error of (*zT*)_TDIS,DAQ_ increased using raw data at a sampling rate of 100 kHz owing to the small SNR in Fig. 3d. We concluded that *zT* = ((*R*_*ohm*_ + *R*_*TE*_)/*R*_*ohm*_ − 1) can be determined by the TDIS method using *R*_*ohm*_ = (*R*_*ohm*_)_IS_ = *Z*_*mea*_(*ω* → ∞) measured by the lock-in amplifier and *R*_*ohm*_ + *R*_*TE*_ = (*R*_*ohm*_ + *R*_*TE*_)_TH,VM_ = *R*_*mea*_(*t* → ∞) measured by the voltmeter within several minutes. Precise measurements were obtained directly using a combination of AC and DC electrometric measurements without any heat measurements.

**Table 1:** A summary of techniques for direct *zT* estimation and its notes.

Technique for *zT* estimation		Impedance spectroscopy (IS)	Transient Harman method (TH)	Pulse and transient Harman method (PTH)	Time domain impedance spectroscopy (TDIS)
(notation)		(*zT*)_IS_	(*zT*)_TH_	(*zT*)_PTH_	(*zT*)_TDIS_
Object domain(s)		Frequency	Time	Time	Frequency and time
*R*_*ohm*_ *Z*_*mea*_(*ω* → ∞) or *R*_*mea*_(*t* → 0)	Required device(s) for data acquisition	*Z*_*mea*_(*ω* → ∞) for lock-in amplifier	*R*_*mea*_(*t* → 0) = (*R*_*ohm*_)_TH_ by DAQ or voltmeter	*R*_*mea*_(*t* → 0) = (*R*_*ohm*_)_pulse_ by DAQ using pulse current	*Z*_*mea*_(*ω* → ∞) for lock-in amplifier
	Requirement(s)	*ω*/*ω*_*R2C*_ > 10^4^ or |*ϕ*|< 0.1°	*R*_*mea*_(*t* → 0) by high frequency data acquisition	*R*_*mea*_(*t* → 0) = (*R*_*ohm*_)_pulse_ at *t*_*p*_/*τ*_*RC*_ < 10^–3^	*ω*/*ω*_*R2C*_ > 10^4^ or |*ϕ*|< 0.1°
*R*_*ohm*_ + *R*_*TE*_ *Z*_*mea*_(*ω* → 0) or *R*_*mea*_(*t* → ∞)	Required device(s) for data acquisition	*Z*_*mea*_(*ω* → 0) for lock-in amplifier or by quasi-AC method^28^	*R*_*mea*_(*t* → ∞) = (*R*_*ohm*_ + *R*_*TE*_)_TH_ by DAQ or voltmeter	*R*_*mea*_(*t* → ∞) = (*R*_*ohm*_ + *R*_*TE*_)_TH_ by DAQ	*R*_*mea*_(*t* → ∞) = (*R*_*ohm*_ + *R*_*TE*_)_TH_ by DAQ or voltmeter
	Requirement(s)	*ω*/*ω*_*R2C*_ < 10^–2^ or |*ϕ*|< 0.1°	*t*/*τ*_*RC*_ > 10	*t*/*τ*_*RC*_ > 10	*t*/*τ*_*RC*_ > 10
Notation of *zT*		$\left( {zT} \right)_{IS} = \frac{{Z_{mea} \left( {\omega \to 0} \right)}}{{Z_{mea} \left( {\omega \to \infty } \right)}} - 1$	$$\begin{gathered} \left( {zT} \right)_{TH} = \frac{{R_{mea} \left( {t \to \infty } \right)}}{{R_{mea} \left( {t \to 0} \right)}} - 1 \\ = \frac{{\left( {R_{ohm} + R_{TE} } \right)_{TH} }}{{\left( {R_{ohm} } \right)_{TH} }} - 1 \\ \end{gathered}$$	$$\begin{gathered} \left( {zT} \right)_{PTH} = \frac{{R_{mea} \left( {t \to \infty } \right)}}{{R_{mea} \left( {t \to 0} \right)}} - 1 \\ = \frac{{\left( {R_{ohm} + R_{TE} } \right)_{TH} }}{{\left( {R_{ohm} } \right)_{pulse} }} - 1 \\ \end{gathered}$$	$$\begin{gathered} \left( {zT} \right)_{TDIS} = \frac{{R_{mea} \left( {t \to \infty } \right)}}{{R_{mea} \left( {t \to 0} \right)}} - 1 \\ = \frac{{\left( {R_{ohm} + R_{TE} } \right)_{TH} }}{{Z_{mea} \left( {\omega \to \infty } \right)}} - 1 \\ \end{gathered}$$
Additional requirements		Optimum current *I*_*max*_(*t* > 0) < < *R*_*ohm*_/|*S*|*T* due to *Q*_*P*_ > > *Q*_*J*_			
		Precise temperature control *ΔT*_*f*_ (< < *ΔT* = *zT R*_*ohm*_*I*_*max*_(*t* > 0)/|*S*|)			
		high vacuum (~ 10^–4^ Pa) to ensure adiabatic condition			
Measurement period		More than several hours	Several minutes	Several minutes	Several minutes
Advantage(s)		Exact	Simple and easy to perform	Simple	Exact, easy, and fast
Disadvantage(s)		Long time required for estimation at *Z*_*mea*_(*ω* → 0)	Not exact due to misinterpretation *R*_*mea*_(*t* → 0)		…

In this study, we reported the *zT* estimation using the TM owing to its higher resistance (~ 1 Ω) compared with a TE (~ 1 to 10 mΩ). In the future, the measurement technique of the TDIS method will be applied to determine *zT* using any TEs with a definite geometry, such as a rectangular solid TE. Furthermore, we plan to establish the accuracy of the TDIS method using the temperature dependence of the *zT* of BiTe, which decreases with temperature in the lower temperature region^12^.

## Conclusions

In this study, we developed a new method to directly estimate *zT*. The proposed method is based on the theory and model of the IS method using frequency domain with a Π-shaped TM for a BiTe system. However, the IS method possesses several drawbacks, such as the long time required to determine *zT*. The reason behind this drawback is that the information about both *Z*_*mea*_(*ω* → ∞) and *Z*_*mea*_(*ω* → 0) using frequency domain with AC is required. In addition, we used the TH method using the time domain with DC, which is based on the time dependence of the resistance *R*_*mea*_(*t*), to measure the resistance *R*_*mea*_(*t* → ∞) and *R*_*mea*_(*t* = 0). However, the results showed that determining *R*_*mea*_(*t* → 0) using the TH method is difficult. Furthermore, we attempted to estimate *R*_*mea*_(*t* → 0) using the PTH method with pulse DC. However, we found that continuous DC was unsuitable for determining *R*_*mea*_(*t* → 0) because determining the resistance derived from the electronic and Peltier heat at *R*_*mea*_(*t* → 0) is difficult. To overcome these drawbacks, we proposed the TDIS method by combining the frequency and time domains to determine *R*_*ohm*_ = *Z*_*mea*_(*ω* → ∞) using the lock-in amplifier with AC and *R*_*ohm*_ + *R*_*TE*_ = *R*_*mea*_(*t* → ∞) using the voltmeter with DC. Finally, *zT* was estimated as *zT* = *R*_*mea*_(*t* → ∞)/*Z*_*mea*_(*ω* → ∞) − 1 using optimum current *I*_*opt*_ that satisfies the condition *Q*_*P*_ (Peltier heat) >  > *Q*_*J*_ (Joule heat), given that *I*_*opt*_ < <|*S*|*T*/*R*_*ohm*_. Furthermore, the estimated *zT* values of the TM using the IS and the TDIS methods were in perfect agreement, i.e., 0.811 at 300 K. Moreover, the TDIS method helped in qualitatively and quantitatively describing *zT* obtained from the IS method. We expect that this study will aid in developing more effective methods to determine *zT* precisely within several minutes for not only TMs but also any given TE.

## Methods

A commercial-base Π-shaped thermoelectric module composed of BiTe was prepared (KSML007F, KELK). The total number (*n*) of the TEs for n- and p-types was *n* = 14. The impedance *Z*_*mea*_(*ω*) and resistance *R*_*mea*_(*t*) were measured by four-probe method after attaching lead-wires to apply current and measure the voltage^16^. One side of the module was tightly fixed by a spring plate to a sample stage capable of controlling the temperature using a precise temperature control system (336, Lakeshore) at 300.000 ± 0.3 mK by calibrated Cernox thermo-sensor (Lakeshore) and two PID feedback heaters chilled by a cryo cooler (RDK-101D, Sumitomo Heavy Industry) under 10^–4^ Pa by vacuum pumps^24,25^. The frequency dependence of *Z*_*mea*_(*ω*) was measured by a lock-in amplifier (SR830, Stanford Research Systems) using an AC source (6221, Keithley) for frequencies higher than 10 mHz. Conversely, the quasi-AC method was employed using a DC source and a voltmeter (2182A, Keithley) for frequencies less than 10 mHz, implemented using a high-precision AC source and digital multimeter using real-time data acquisition for the low-frequency region^12,28^. The time dependence of the resistance *R*_*mea*_(*t*) was measured using a DC and pulse current source (6221, Keithley) for currents less than 100 mA, and a DC source (2400, Keithley), voltmeter (2182A, Keithley), and DAQ system (USB-6281, NI) for currents higher than 100 mA. All the instruments were connected through GPIB and USB cables and controlled appropriately by the LabVIEW (NI) program.

## Supplementary Information

Supplementary Information.

## Data Availability

Data is available upon reasonable request to the corresponding author.

## List of symbols

TE: Thermoelectric element
TM: Thermoelectric module
AC: Alternating current
DC: Direct current
IS: Impedance spectroscopy
TH: Transient Harman method
PTH: Pulse and transient Harman method
TDIS: Time domain impedance spectroscopy
R2C model: A simple model of thermoelectric or thermo-module using two resistance and one capacitance
RC model: A simplified model of thermoelectric or thermo-module using two resistance and one capacitance
VM: Voltmeter
DAQ system: Data acquisition system
SNR: Signal-to-noise ratio
*n*: Number of elements in a thermoelectric module (TM) (–)
*T*: Absolute temperature (K)
*Δ**T*: Temperature difference (K)
*Δ**T*_*f*_: Temperature fluctuation of sample stage anchoring (K)
*S*: Seebeck coefficient (V/K)
*ρ*: Resistivity (Ωm)
*κ*: Thermal conductivity (W/mK)
*ω*: Angular frequency (rad/s)
*ω*_*TE*_: Characteristic frequency of thermoelectric or thermo-module using impedance spectroscopy (IS) (rad/s)
*ω*_*R2C*_: Characteristic frequency of thermo-module using R2C approximation (rad/s)
*Z*(*ω*): Impedance as a function of angular frequency at angular frequency *ω* (Ω)
*Z*_*R2C*_(*ω*): Impedance as a function of angular frequency at angular frequency *ω* applying R2C approximation (Ω)
*Z*_*mea*_(*ω*): Measured impedance as a function of angular frequency at angular frequency *ω* (Ω)
*R*_*ohm*_: Ohmic resistance (Ω)
*R*_*AC*_: AC resistance with an alternating current by Harman method (Ω)
*R*_*DC*_: DC resistance with a direct current by Harman method (Ω)
*R*_*TE*_: Thermoelectric resistance (Ω)
*R*_*ohm*_ + *R*_*TE*_: Sum of ohmic (*R*_*ohm*_) and thermoelectric (*R*_*TE*_) resistances (Ω)
*C*_*TE*_: Thermoelectric capacity in system (F)
*t*: Time (s)
*τ*_*exp*_: Expected heat time constant in system (s)
*τ*_*R2C*_: Estimated time constant using R2C approximation in Eq. (3) (s)
*τ*_*RC*_: Estimated time constant using RC approximation in Eq. (4) (s)
*T*_*p*_: Pulse period of pulse and transient Harman (PTH) method (s)
*t*_*p*_: Pulse width of pulse and transient Harman (PTH) method (s)
*R*(*t*): Resistance as a function of time *t* (Ω)
*R*_*mea*_(*t*): Measured resistance as a function of time *t* (Ω)
(*R*_*ohm*_)_IS_: Estimated ohmic resistance (*R*_*ohm*_) by impedance spectroscopy (IS) method (Ω)
(*R*_*ohm*_)_TH,DAQ_: Estimated ohmic resistance (*R*_*ohm*_) by transient Harman (TH) method using data acquisition (DAQ) system (Ω)
(*R*_*ohm*_)_TH,DAQ,R2C_: Estimated ohmic resistance (*R*_*ohm*_) by transient Harman (TH) method using data acquisition (DAQ) system assuming R2C approximation in Eq. (3) (Ω)
(*R*_*ohm*_)_TH,DAQ,RC_: Estimated ohmic resistance (*R*_*ohm*_) by transient Harman (TH) method using data acquisition (DAQ) system assuming RC approximation in Eq. (4) (Ω)
(*R*_*ohm*_)_pulse_: Estimated ohmic resistance (*R*_*ohm*_) by pulse DC using data acquisition (DAQ) system (Ω)
(*R*_*ohm*_)_delta_: Estimated ohmic resistance (*R*_*ohm*_) using delta method, in which a current source is synchronized with an oscillating square wave at a frequency using a voltmeter (Ω)
(*R*_*ohm*_ + *R*_*TE*_)_IS_: Sum of estimated ohmic and thermoelectric resistances (*R*_*ohm*_ + *R*_*TE*_) measured by impedance spectroscopy (IS) method (Ω)
(*R*_*ohm*_ + *R*_*TE*_)_TH,VM_: Sum of estimated ohmic and thermoelectric resistances (*R*_*ohm*_ + *R*_*TE*_) measured by transient Harman (TH) method using voltmeter (VM) (Ω)
(*R*_*ohm*_ + *R*_*TE*_)_TH,DAQ_: Sum of estimated ohmic and thermoelectric resistances (*R*_*ohm*_ + *R*_*TE*_) measured by transient Harman (TH) method using data acquisition (DAQ) system (Ω)
(R_ohm_ + R_TE_)_TH,DAQ,R2C_: Sum of estimated ohmic and thermoelectric resistances (*R*_*ohm*_ + *R*_*TE*_) measured by transient Harman (TH) method using data acquisition (DAQ) system assuming R2C approximation in Eq. (3) (Ω)
(R_ohm_ + R_TE_)_TH,DAQ,RC_: Sum of estimated ohmic and thermoelectric resistances (*R*_*ohm*_ + *R*_*TE*_) measured by transient Harman (TH) method using data acquisition (DAQ) system assuming RC approximation in Eq. (4) (Ω)
*z*: Figure of merit (= *S*^2^/*ρκ*) (1/K)
*zT*: Dimensionless figure of merit (= *zT* = *S*^2^*T*/*ρκ* = *R*_*TE*_/*R*_*ohm*_ = (*R*_*ohm*_ + *R*_*TE*_)/*R*_*ohm*_ − 1) (–)
(*zT*)_IS_: Estimated dimensionless figure of merit (*zT*) by impedance spectroscopy (IS) (–)
(*zT*)_TH,DAQ_: Estimated dimensionless figure of merit (*zT*) by transient Harman (TH) method using data acquisition (DAQ) system (= (*R*_*ohm*_ + *R*_*TE*_)_TH,DAQ,RC_/(*R*_*ohm*_)_TH,DAQ,RC_ − 1) (–)
(*zT*)_PTH_: Estimated dimensionless figure of merit (*zT*) by pulse and transient Harman (PTH) method using data acquisition (DAQ) system (= (*R*_*ohm*_ + *R*_*TE*_)_TH,DAQ_/(*R*_*ohm*_)_pulse_ − 1) (–)
(*zT*)_TDIS,VM_: Estimated dimensionless figure of merit (*zT*) by time domain impedance spectroscopy (TDIS) method using voltmeter (VM) (= (*R*_*ohm*_ + *R*_*TE*_)_TH,VM_/(*R*_*ohm*_)_IS_ − 1) (–)
(*zT*)_TDIS,DAQ_: Estimated dimensionless figure of merit (*zT*) by time domain impedance spectroscopy (TDIS) method using data acquisition (DAQ) system applying RC approximation (= (*R*_*ohm*_ + *R*_*TE*_)_TH,DAQ,RC_/(*R*_*ohm*_)_IS_ − 1) (–)
*I*(*t*): Passing current [A] for DC or [A_rms_] for AC at time *t*
*I*_*opt*_: Optimum current for measurement (< <|*S*|*T*/*R*_*ohm*_) [A] or [A_rms_]
*I*_*max*_: Maximum suitable current for time domain impedance spectroscopy (TDIS) [A] or [A_rms_]
*Q*_*P*_: Peltier heat ( =|*S*|*TI*) (W)
*Q*_*J*_: Joule heat (= *R*_*ohm*_*I*_2_) (W)
*A*: Cross-sectional area of thermoelectric material (m^2^)
*L*: Length of thermoelectric material (m)
*η*: A proportional factor associated with heat flow of Peltier heat (*Q*_*J*_) to one side of the thermoelectric element (–)
*q*_*P*_: Peltier heat flux through each thermoelectric element (= *Q*_*P*_/*A*) (W/m^2^)
*α*: Thermal diffusivity (m^2^/s)
*ϕ*: Phase angle of impedance (= tan^−^^1^(Im[*Z*_*mea*_(*ω*)]/Re[*Z*_*mea*_(*ω*)]) (rad)
