# Supplementary material for: Proposal of time domain impedance spectroscopy to determine precise dimensionless figure of merit for thermoelectric modules within minutes
Source: Sci Rep. 2022 Jul 13;12:11967. doi: 10.1038/s41598-022-15947-4 (PMC9279445; doi:10.1038/s41598-022-15947-4)
Supplement: Supplementary file 1 — Supplementary Information. [file 41598_2022_15947_MOESM1_ESM.docx]

**Supplementary Information**

**Proposal of Time Domain Impedance Spectroscopy to determine Precise Dimensionless Figure of Merit for Thermoelectric Modules within Minutes**

**Yasuhiro Hasegawa**^1,2^ **and Mai Takeuchi**^1^

^1^ Graduate School of Science and Engineering, Saitama University, 255, Shimo-okubo, Sakura, Saitama, 338-8570, Japan

^2^ [hasegawa@mail.saitama-u.ac.jp](mailto:hasegawa@mail.saitama-u.ac.jp)

Figure S1a shows the switching frequency of square wave current (*f_s_*) dependence of estimated (*R_ohm_*)_delta_ for each scan rate *t_scan_* by delta mode passing ±1 mA.^1^ The (*R_ohm_*)_delta_ at *f_s_* = 1 Hz was overestimated because the measured resistance *R_mea_*(*t* → 0) was added to *zT*×*t*/*τ_RC_* from *R_ohm_* using the RC approximation given below:

$R_{mea}(t\to0)\simeq R_{RC}(t\to0)=R_{ohm}+R_{TE}\left(1-\exp\left\{-\frac{t}{\tau_{RC}}\right\}\right)\simeq R_{ohm}+R_{TE}\frac{t}{\tau_{RC}}=R_{ohm}\left(1+zT\frac{t}{\tau_{RC}}\right)$. (S1)

At *t* = *t_p_*, (*R_ohm_*)_delta_ would be taken as (*R_ohm_*)_delta_ = {*R_mea_*(0) + *R_mea_*(*t_p_*)}/2 using simple calculation. At *f_s_* = 1 Hz (pulse width *t_p_* = 1/2*f_s_* = 0.5 s), (*R_ohm_*)_delta_ = {480.0 + 480.0 × (1+ 0.811 × 0.5/4.06)}/2 = 504.0 mΩ. This value is similar to the estimated (*R_ohm_*)_delta_ at *f_s_* = 1 Hz. However, to decrease the contribution of *t*/*τ_RC_*, a considerably higher *f_s_* is required. At higher *f_s_*, (*R_ohm_*)_delta_ approaches (*R_ohm_*)_IS_ using *t_scan_* = 0.2 ms, which is the highest scan rate of the measurement. If the combination between *f_s_* and *t_scan_* is unsuitable, (*R_ohm_*)_delta_ decreases rapidly because skip reading of data occurs, as shown in the inset in Fig. S1. For instance, at *t_scan_* = 100 ms, skip reading increased to *f_s_* > 1 Hz. When a suitable combination of *f_s_* and *t_scan_* is selected, the estimated (*R_ohm_*)_delta_ would approach (*R_ohm_*)_IS_; however, determining the suitable *f_s_* and *t_scan_* for each measurement is difficult.

Figure S1b also shows the scan rate (*t_scan_*) dependence of the estimated (*R_ohm_*)_delta_ at optimum switching frequency of square wave current (*f_s_*) from Fig. S1a. At *t_scan_*/*τ_RC_* < 10^-3^, (*R_ohm_*)_delta_ approaches (*R_ohm_*)_IS_, as expected from Eq. S1. From the investigations, the measurable switching frequency *f_s_* increased up to 17 Hz, as shown in the inset in Fig S1b; however, the influences of the skip reading of the data and low path filter in the multi-meter could cause certain measurement errors.

**References**

1. Low Level Measurement Handbook- 7^th^ Edition, Keithley/Tektronix, <https://download.tek.com/document/LowLevelHandbook_7Ed.pdf>


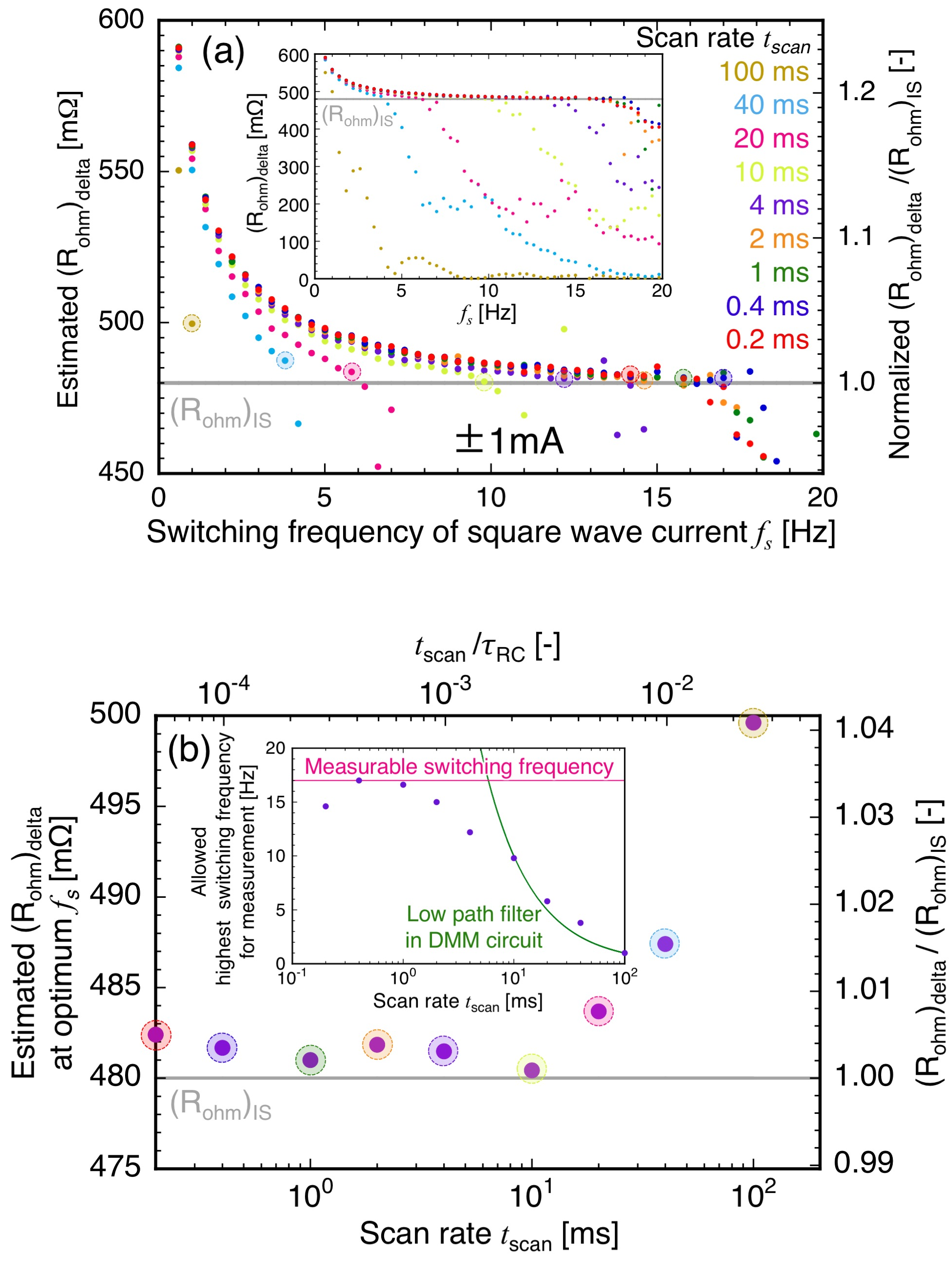


**Supplementary Figure S1.** (a) Switching frequency of square wave current (*f_s_*) dependence of estimated (*R_ohm_*)_delta_ for each scan rate *t_scan_* by delta mode. Inset shows apparent estimated (*R_ohm_*)_delta_ from 0 to 600 mΩ. (b) Scan rate *t_scan_* dependence of (*R_ohm_*)_delta_ at optimum frequency *f_s_* from Fig. S1a. Inset shows permitted highest measurable switching frequency, *f_s_*, from the measurement at each *t_scan_*.
